# Protective immunity against influenza H5N1 virus challenge in chickens by oral administration of recombinant *Lactococcus lactis* expressing neuraminidase

**DOI:** 10.1186/s12917-015-0399-4

**Published:** 2015-04-02

**Authors:** Han Lei, Xiaojue Peng, Jiexiu Ouyang, Daxian Zhao, Huifeng Jiao, Handing Shu, Xinqi Ge

**Affiliations:** School of Medicine, Southwest Jiaotong University, Chengdu, 6111756 China; Department of Biomedical Engineering, State University of New York, Binghamton, 13902 USA; Department of Biotechnology, College of Life Science, Nanchang University, Jiangxi, 330031 China

**Keywords:** *L.lactis*/pNZ2103-NA, H5N1 virus, Protective immunity

## Abstract

**Background:**

Highly pathogenic H5N1 avian influenza viruses pose a debilitating pandemic threat in poultry. Current influenza vaccines predominantly focus on hemagglutinin (HA) which anti-HA antibodies are often neutralizing, and are used routinely to assess vaccine immunogenicity. However, Neuraminidase (NA), the other major glycoprotein on the surface of the influenza virus, has historically served as the target for antiviral drug therapy and is much less studied in the context of humoral immunity. The aim of this study was to evaluate the protective immunity of NA based on *Lactococcus lactis* (*L.lactis*) expression system against homologous H5N1 virus challenge in a chicken model.

**Results:**

*L.lactis*/pNZ2103-NA which NA is derived from A/Vietnam/1203/2004 (H5N1) (VN/1203/04) was constructed based on *L.lactis* constitutive expression system in this study. Chickens vaccinated orally with 10^12^ colony-forming unit (CFU) of *L.lactis*/pNZ2103-NA could elicit significant NA-specific serum IgG and mucosa IgA antibodies, as well as neuraminidase inhibition (NI) titer compared with chickens administered orally with saline or *L.lactis*/pNZ2103 control. Most importantly, the results revealed that chickens administered orally with *L.lactis*/pNZ2103-NA were completely protected from a lethal H5N1 virus challenge.

**Conclusions:**

The data obtained in the present study indicate that recombinant *L.lactis*/pNZ2103-NA in the absence of adjuvant can be considered an effective mucosal vaccine against H5N1 infection in chickens via oral administration. Further, these findings support that recombinant *L.lactis*/pNZ2103-NA can be used to perform mass vaccination in poultry during A/H5N1 pandemic.

## Background

Rapid worldwide dissemination of highly pathogenic avian influenza (HAPI) H5N1 viruses among poultry and ongoing viral evolution through genetic drift and reassortment raise concerns of a potential influenza pandemic [1]. HAPI H5N1 virus has emerged in Southeast Asian and resulted in the destruction of millions of birds [2]. Concerns about the potential for the generation of a pandemic H5 strain and its concomitant morbidity and mortality are spurring the search for an effective vaccine. Vaccination is the most safe and effective way to prevent and control H5N1 infection in poultry. Currently, two commercial inactivated H5N1vaccines (Re-1 and Re-5) have been widely applied in domestic duck in many Asian countries [3]. However, these approved vaccines against H5N1 viruses produced in fertilized eggs have serious limitations, particularly the limited capability of producing conventional inactivated influenza H5N1 vaccines could severely hinder the ability to control the pandemic spread of avian influenza through vaccination [1,4]. In addition, conventional vaccines utilizing the hemagglutinin (HA) of H5N1 viruses have been poor immunogenicity and have safety issues [4]. Although novel approaches, such as recombinant DNA vaccines and virus-like particles (VLPs), show some promising signs against H5N1 infection in mice or poultry [5-8], the risk of generating a reassortant prohibit the use of this vaccine in most instances. Therefore, there is a clear need for a new vaccine strategy in poultry that provides increasing immunogenicity and safety.

For mucosal immunization, lactic acid bacterium (LAB) is more attractive vaccine delivery system than other live vaccine vehicles, such as *Shigella*, *Salmonella*, and *Listeria* [9-11]. *Lactococcus lactis* (*L.lactis*), a typical model of lactic acid bacteria, is an ideal vaccine delivery vector and has been engineered to express many viral antigens [12,13]. It was shown previously that *L.lactis*, expressing hemaglutinin (HA) from A/chicken/Henan/12/2004(H5N1) and then coated by enteric capsule, is a safe and effective vaccine against avian influenza H5N1 virus infection in mice [14]. Similarly, it was described that HA1 from A/chicken/Henan/12/2004(H5N1) virus was displayed on the surface of *L.lactis*, and showed it to be protective against homologous H5N1 virus by oral co-administration with CTB in mice [15]. Recently, it also shown that intranasal immunization of *L.lactis*-HA combined with mucosal adjuvant LTB could provide protection against homologous H5N1 in chickens [16]. However, most of these vaccines focus on raising a humoral response against hemagglutintin (HA) of H5N1 viruses. Neuraminidase (NA) is another major glycoprotein on the surface of the virus and has historically served as the target for antiviral drug therapy and is much less studied in the context of humoral immunity [17]. It remains largely unknown regarding the immunogenicity of recombinant *L.lactis* expressing neuraminidase (NA) in poultry via oral administration.

In the present study, we develop a constitutive expression system by constructing recombinant *L.lactis* expressing NA gene from A/Vietnam/1203/2004 (H5N1) (VN/1203/04) and then evaluating its immunogenicity via oral administration without the use of adjuvant in a chicken model. This study reported here suggests that this system can be used as a platform technology to develop a mucosal NA vaccine for preventing and controlling H5N1 infection in poultry.

## Methods

### Construction of plasmid expressing NA and expression on *L.lactis*

The NA gene (1459 bp) of A/Vietnam/1203/2004 (H5N1) was PCR-amplified from pCDNA3.1-HA (kindly provided by St. Jude Children’s Research Hospital, Memphis, TN, USA) using the following primers: NA-F: CTA***GCTAGC***GGTACCGCCGCCACCATGAA (*Nhe* I); NA-R: CCG***AAGCTT***ACAGGAAGTATTCAATC (*Hind* III) and cloned into *L.lactis* based constitutive expression plasmid pNZ2103 (purchased from MoBiTec, Goettingen, Germany), the resulting plasmid was transformed into competent *L.lactis* NZ3000, the positive clone was named as *L.lactis*/pNZ2103-NA.

Western blot analysis was described previously [14] and *L.lactis*/pNZ2103 was used as a negative control.

### Animal experiments and sample collection

For oral administration of chickens, 7-day-old specific-pathogen-free (SPF) single comb white leghorn chickens from an in-house flock (Institute of Jiangxi Agriculture, China) were used in this study. The concentration of recombinant *L.lactis*/pNZ2103-NA was adjusted to 10^12^ colony forming unit (CFU)/ml with sterile saline.

Three groups of 16 chickens each were immunized with oral administration of 1 ml of sterile saline, 10^12^ CFU of *L.lactis*/pNZ2103 or 10^12^ CFU of *L.lactis*/pNZ2103-NA, respectively. Prime immunization was performed at day 0, 1, 2, 3 and boosted at day 17, 18, 19, 20.

At day 15 and day 34 after the first immunization, blood samples were collected from the retro-orbital plexus. Sera were separated by centrifugation of blood at 2,000 × g for 10 min and stored at -20°C until use. Intestine and upper respiratory were isolated from the vaccinated chickens and washed with 500 μL sterile saline, respectively.

At two weeks after the last immunization, chickens were lightly anesthetized with CO_2_ and inoculated intranasally with 25 μl of 10^4^ EID_50_ of VN/1203/04 virus through the choanal slit to determine protection efficacy. Chickens were observed for illness, weight loss, and death for 14 days after H5N1 virus infection. H5N1 virus challenge experiments must be strictly performed under the enhanced bio-safety level-3 laboratory (BSL-3).

The chickens were managed with pelleted feed and sterile water, maintained in a SPF environment and all efforts were made to minimize suffering following approval from the Institute Animal Care and Use Committee of the Nanchang University (Approval No. 726-14).

### Enzyme-linked immunosorbent assay (ELISA)

Immune sera from the vaccinated chickens were collected by bleeding from the wing vein and treated with receptor-destroying enzyme from *Vibrio cholerae* (Denka-Seiken, San Francisco, CA) before being tested for the presence of H5-specific antibodies as described previously [16].

NA-specific immunoglobulin G (IgG) and secretory immunoglobulin A (IgA) antibodies were detected by enzyme-linked immunosorbent assay (ELISA) using recombinant NA protein as a coating antigen as described previously [14]. ELISA end point titers were expressed as the highest dilution that yielded an optical density greater than twice the mean plus one standard deviation of that of similarly diluted negative control samples.

### Neuraminidase inhibition (NI) assay

The anti-NA immune response was evaluated by Bioluminescence-based neuraminidase inhibition kit. To perform this, 50 μl of chickens sera from each group was taken at 1/2 dilutions which were half diluted further till 1/1024 in a 96-well micro-titer plate. 50 μl of purified rNA (0.25 mg/ml) was added to each well and incubated at 37°C for 2 h. The neuraminidase inhibition titer was represented as the highest dilution until there was no neuraminidase activity observed.

### Data analysis

Data are presented as the means ± standard deviations (S.D.) and are representative of at least three independent experiments. All analysis for statistically significant differences was performed by the Student *t* test and one-way ANOVA. A *p* value less than 0.05 was considered to be significant.

## Results

### Expression of NA protein on *L.lactis*

**Figure 1:**
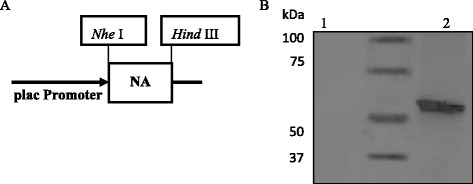
**Construction of pNZ2103-NA and expression of NA protein on** ***L.lactis*** **. (A)** A schematic diagram of constitutive plasmid pNZ2103-NA. **(B)** Western blot analysis of recombinant *L.lactis*/pNZ2103-NA expression. Lane 1: negative control *L.lactis*/pNZ2103; Lane 2: MagicMark™ XP Western Protein Standard; Lane 3: *L.lactis*/pNZ2103-NA. A specific protein band of around 54 kDa corresponding to NA was detected using anti-NA monoclonal antibody.

In this study, we generated a constitutive plasmid pNZ2103-NA containing NA gene from A/Vietnam/1203/2004 (H5N1) (Figure 1A). Expression of NA protein on *L.lactis* NZ3000 was confirmed by western blotting using anti-HA monoclonal antibody (Figure 1B). As we expected, there is no band shown in the *L.lactis*/pNZ2103 cells, while a specific band was observed at expected size for NA protein (approximately 54 kDa) (Figure 1B, Lane 3) in the *L.lactis*/pNZ2103-NA cells.

### Immune responses elicited d by oral administration of *L.lactis*/pNZ2103-NA

**Figure 2:**
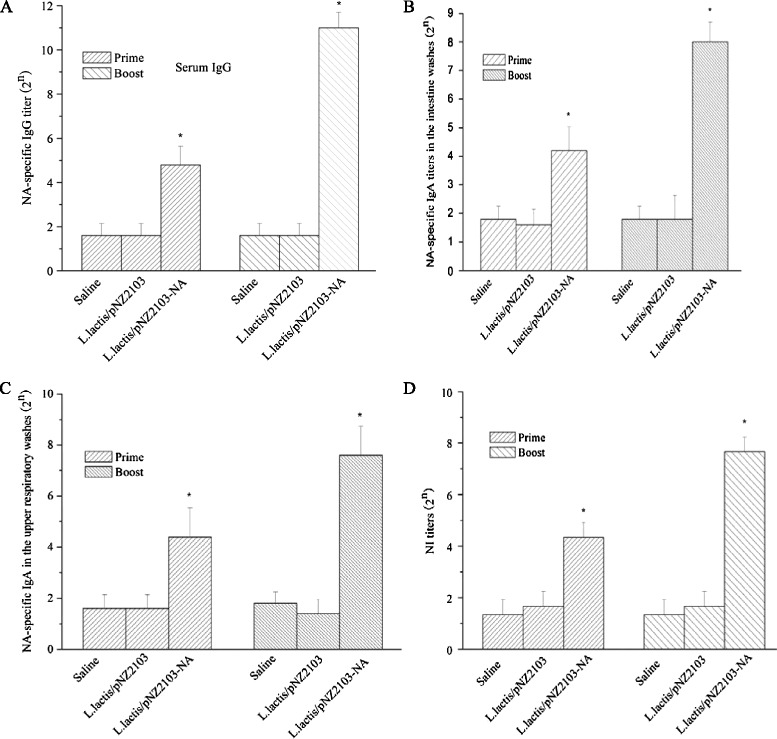
**Oral administration of recombinant** ***L.lactis*** **/pNZ2103-NA induces NA-specific immune responses in chickens.** Chickens were immunized orally with saline, *L.lactis*/pNZ2103 or *L.lactis*/pNZ2103-NA at day 0, 1, 2, 3 and day 17, 18, 19, 20. Sera (n = 16/group), intestine (n = 3/group) and upper respiratory (n = 3/group) washes were collected at day 15 and day 34 after the prime immunization. **(A)** NA-specific IgG antibody was measured by ELISA in the sera. **(B)** NA-specific IgA antibody was assessed in the intestinal washes. **(C)** NA-specific IgA antibody was assessed in the upper respiratory washes. **(D)** NI titers were determined using rNA protein. Data are presented as the means ± standard deviations (S.D.). The asterisk indicates a significant difference between *L.lactis*/pNZ2103-NA and other groups (saline or *L.lactis*/pNZ2103) (* *p* < 0.05).

At day 15 and day 34 after the prime immunization, sera samples were obtained from all chickens to screen for antibody responses as a marker of immunogenicity. There was no significant serum IgG detected between *L.lactis*/pNZ2103-NA and saline or *L.lactis*/pNZ2103 group at day 15 after the prime immunization. However, at day 34 after the prime immunization, chickens administered orally with *L.lactis*/pNZ2103-NA could elicit a higher significant NA-specific IgG titer than other groups (saline or *L.lactis*/pNZ2103) (Figure 2A).

To assess the mucosal immune responses, the secretory mucosal IgA levels were determined by ELISA. Intestinal and upper respiratory washes were also collected at day 15 and day 34 after the prime immunization. As shown in Figure 2B and C, *L.lactis*/pNZ2103-NA induced significantly increased levels of NA-specific mucosal IgA compared to saline or *L.lactis*/pNZ2103 group at day 34 after the prime immunization in the intestinal and upper respiratory washes. These results are consistent with the detection of serum IgG antibody.

These data demonstrate that chickens vaccinated orally with *L.lactis*/pNZ2103-NA after prime-boost immunization can result in significant IgG and IgA levels which may contribute to protection against virus infection.

### Neuraminidase inhibition (NI) titers induced by *L.lactis*/pNZ2103-NA

Similarly, oral vaccination with *L.lactis*/pNZ2103-NA induced a higher NI titer compared to other groups (saline or *L.lactis*/pNZ2103) (Figure 2D). These results support that recombinant *L.lactis*/pNZ2103-NA is immunogenic without the use of adjuvant in a chicken model.

### Protection efficacy against H5N1 challenge

**Figure 3:**
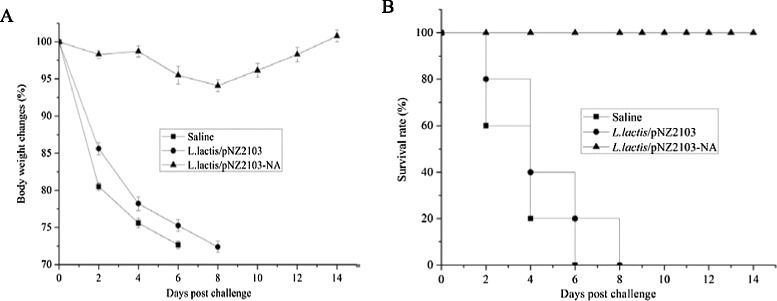
**Protection efficacy of** ***L.lactis*** **/pNZ2103-NA against H5N1 virus challenge. (A)** Weight changes as a percentage. **(B)** Survival rate. (n = 10 per group).

At two weeks after the last immunization, all chickens were challenged by intranasal inoculation with 25 μl of 10^4^ EID_50_ of VN/1203/04 (H5N1). All chickens immunized orally with saline or *L.lactis*/pNZ2103 experienced substantial weight loss beginning at day 2 post challenge and death by 6 to 8 days post infection. In contrast, chickens immunized orally with *L.lactis*/pNZ2103-NA showed only mild and transient loss of body weight and survived the lethal challenge (Figure 3).

## Discussion

Vaccination is an integral component of strategies aiming to prevent and control pandemic influenza in poultry. Unfortunately, current commercial inactivated influenza H5N1 vaccines for poultry are generated from the whole viruses that have serious safety issues and poor immunogenic [8,18]. Therefore, it is crucial to develop a safe and effective influenza H5N1 vaccine that can be applied broadly and rapidly in poultry during H5N1 pandemic. Our previous study has shown that intranasal immunization of *L.lactis*-HA combined with LTB can provide protective immunity in chicken [16]. However, most of influenza H5N1 vaccines focus on HA, it remains unclear that whether NA express on *L.lactis* has immunogenicity and poses potential for H5N1 vaccine development in poultry via oral administration. Further, it is well recognized in the NA field that a vaccine that purely raises antibodies to neuraminidase is not desirable and would not be as effective as one which includes some combination of the NA and HA antigen [19]. Here, we hypothesize that recombinant *L.lactis* expressing NA can confer protective immunity against H5N1 challenge. To address this hypothesis, oral administration of *L.lactis*/pNZ2103-NA in the absence of adjuvant could induce protective immunity against H5N1 infection in a chicken model. This provides an evidence that *L.lactis*/pNZ2103-NA can serve as an effective mucosal vaccine to prevent and control H5N1 infection in poultry without the use of adjuvant.

The mucosal immune system is the first immunological barrier against the pathogens that invade the body via the mucosal surface. Thus, the induction of mucosal immunity via mucosal administration (oral or intranasal) is necessary to ensure protection against multiple subtypes of influenza A virus. Secretion of IgA is a representative antibody of mucosal immune response, and confers efficient protection against acquired mucosal infection [20]. It is an effective way to construct the incorporation of viral antigen to recombinant *L.lactis* that is considered essential to boost the interaction of the vaccine with the mucosal immune system [21]. This study revealed that chickens vaccinated orally with optional dosage as 10^12^ CFU of *L.lactis*/pNZ2103-NA was able to induce a significantly higher level mucosal IgA antibody in intestinal and upper respiratory washes (Figure 2B and C). Similarly, chickens administered orally with *L.lactis*/pNZ2103-NA also elicited a higher HA-specific IgG titer and NI titer which played an important role in providing protection against H5N1 lethal infection (Figure 2A and D). Collectively, these results support that *L.lactis*/pNZ2103-NA has a strong immunogenicity via oral immunization route without the use of adjuvant. In this regard, *L.lactis*/pNZ2103-NA can be considered an effective influenza H5N1 vaccine candidate for poultry.

The final protective immunity is most important for vaccine development [22]. Monitoring after post infection of H5N1 virus indicated that there was no significant decrease in the body weight of chickens vaccinated orally with *L.lactis*/pNZ2103-NA. In addition, the survival rate revealed that *L.lactis*/pNZ2103-NA could provide complete protection efficacy against highly pathogenic avian influenza H5N1 virus (Figure 3B). These findings suggest that *L.lactis*/pNZ2103-NA can be considered an effective influenza H5N1 vaccine candidate for mass vaccination in poultry. In addition, influenza vaccines based on *L.lactis* expression system have no safety issues, which make this technology has the potential of becoming one of the most promising platforms for avian influenza H5N1 vaccine development in poultry via oral vaccination. Our long-term goal is to translate these animal studies to preclinical studies, and determine the immunogenicity of recombinant *L.lactis* based vaccines in human, and to augment this technology to develop influnenza universal vaccines against different influenza virus subtypes.

## Conclusion

In conclusion, our findings strongly support oral administration of chickens with *L.lactis*/pNZ2103-NA in the absence of adjuvant can induce significant humoral and mucosal immune responses, as well as NI titers in chickens. Given the induction of protective immunity in the vaccinated chickens, widespread immunization of *L.lactis*/pNZ2103-NA in susceptible poultry would likely provide a significant barrier to the spread of H5N1 virus and also be economically advantageous. Thus, *L.lactis*/pNZ2103-NA may be a promising avian influenza H5N1 vaccine candidate for poultry in the event of the pandemic spread of H5N1 virus.
